# Same-Day Coordination of Mohs Surgery, Lymphoscintigraphy, and Sentinel Lymph Node Biopsy for Merkel Cell Carcinoma: A Single-Center Feasibility Report

**DOI:** 10.3390/cancers18193187

**Published:** 2026-10-02

**Authors:** Victoria H. Ruesch, Justin Thrush, James F. Bena, Claudia Ricotti, Melissa Piliang, Melissa McEnery-Stonelake, Basia Marie Michalski-McNeely, Jon Meine, Jennifer Lucas, Christine Poblete-Lopez, Shlomo Koyfman, Brian Gastman, Allison T. Vidimos

**Affiliations:** 1 School of Medicine, Case Western Reserve University, Cleveland, OH 44106, USA; 2 Department of Dermatology, Cleveland Clinic, Cleveland, OH 44195, USAricottc2@ccf.org (C.R.); pilianm@ccf.org (M.P.); mcenerm@ccf.org (M.M.-S.); meinej@ccf.org (J.M.); lucasj@ccf.org (J.L.); lopezc@ccf.org (C.P.-L.); 3 Department of Quantitative Health Sciences, Cleveland Clinic, Cleveland, OH 44196, USA; benaj@ccf.org; 4 Department of Dermatology, Washington University in St. Louis, St. Louis, MO 63130, USA; basiammichalski@wustl.edu; 5 Department of Radiation Oncology, Cleveland Clinic, Cleveland, OH 44195, USA; koyfmas@ccf.org; 6 Department of Plastic Surgery, Cleveland Clinic, Cleveland, OH 44106, USA; brian.gastman@iovance.com

**Keywords:** Merkel cell carcinoma, Mohs surgery, sentinel lymph node biopsy, care coordination, multidisciplinary care

## Abstract

**Background/Objectives**: Merkel cell carcinoma (MCC) is an aggressive neuroendocrine tumor, most commonly occurring on the head and neck. Incidence of MCC is rising with the aging population. Five-year overall survival (OS) for localized MCC is 50.6%; 5-year OS for patients with distant metastases is 13.5%. The purpose of this study is to determine if coordination of care for the treatment of MCC is logistically feasible, and provide a description of the patient population observed at this institution. **Methods**: All patients with MCC diagnosis from January 2012–June 2024 were identified at our tertiary care center. All patients with MCC were added to our non-melanoma skin cancer registry. Outcomes were compared between those treated with our “Merkel cell carcinoma in a day” (MIAD) protocol and those receiving the standard treatment protocol. **Results**: No significant differences were detected in the recurrence rate, transit metastasis, distant metastasis, and disease-specific death. Nodal metastasis was higher in the MIAD group (HR 4.94, 95% CI 1.08–22.6, *p* = 0.039, six events total). Both groups had similar times to treatment and treatment within 30 days of diagnosis. **Conclusions**: MCC is a rare and aggressive tumor, often exhibiting subclinical tumor spread. Mohs micrographic surgery ensures 100% peripheral and deep margin assessment, with potential for tissue sparing. With thoughtful coordination, lymphoscintigraphy can be completed on the same day as Mohs surgery followed by sentinel lymph node biopsy (SLNB) and reconstructive surgery.

## Simple Summary

In this study, we aimed to test our protocol for the treatment of Merkel cell carcinoma (MCC). Our protocol involves coordination with the Department of Dermatology, the Department of Plastic Surgery, and the Department of Nuclear Medicine so that both Mohs surgery and lymph node biopsy are completed on the same day. We did not detect differences in most outcomes between the two protocols, although the study was small and follow-up was limited. Our findings suggest the same-day protocol is logistically feasible and warrants prospective study.

## 1. Introduction

Merkel cell carcinoma (MCC) is a rare cutaneous malignancy with approximately 700–2000 cases diagnosed annually [1,2,3]. The five-year survival rate for localized disease is 50.6% and 13.5% for those with distant metastasis [4]. The incidence of MCC has been growing in both the United States and globally [3,5]. Further research focused on improved diagnosis and prompt treatment is essential.

There are two pathogenic mechanisms associated with MCC, sun exposure and Merkel cell polyomavirus (MCV) exposure. MCV exposure accounts for about 50–80% of MCC and is associated with improved outcomes [6]. Like other skin cancers, MCC can develop through the accumulation of mutations from UV damage. Numerous mutations have been associated with MCV-negative MCC, including RB, p53, NOTCH1/2, ATM, BRCA1/2, MSH2, BCOR, KMT2A/C/D, ASXL1, ARID1A/B, and SMARCA4 [7]. MCV induces tumorigenesis through the inhibition of retinoblastoma protein (Rb), via the large T antigen (LTA) protein within the viral genome. MCV also codes for a small T antigen (STA) that helps induce viral replication and stabilize the LTA, aiding in tumor formation [8]. Both MCV-positive and MCV-negative MCC have been associated with mutations in receptor tyrosine kinases and the PI3K-AKT-mTOR growth signaling pathway, further enhancing tumor formation and cancer growth [7].

Clinically, MCC often occurs as a rapidly growing, asymptomatic, solitary erythematous or violaceous papule, nodule, or plaque on sun-exposed areas. MCC is most often present in patients who are immunosuppressed or over the age of 50 [9,10]. It can commonly be confused with a benign cyst, dermatofibroma, or squamous cell carcinoma (SCC) often delaying diagnosis [8]. Diagnosis is made with skin biopsy, and imaging should be used to assess for distant metastasis [9].

MCC is typically located in the dermis, different from the non-pathological Merkel cells in the basal layer of the epidermis. Using immunostaining, MCCs are positive for low-molecular-weight keratin and neurofilaments, especially cytokeratin type 20. Positive staining for CK20 and neurofilaments with negative thyroid transcription factor-1 (TTF-1) are the important diagnostic criteria for MCC [11].

Staging of MCC is done according to the American Joint Committee on Cancer (AJCC) Cancer Staging Manual and based on tumor size (T), lymph node invasion (N), and the presence of distant metastasis (M) [9]. The recurrence rate for MCC is significantly higher than that of other skin cancers, especially MCCs of the head and neck which tend to metastasize early and recur more frequently [12]. Recurrence of MCC often occurs within 3 years of initial diagnosis [13]. Newly emerging evidence also suggests circulating tumor DNA may be helpful in determining the recurrence risk [14].

MCC is treated with a variety of approaches depending on the stage and the patient’s comorbidities. Currently, wide resection with 1–2 cm free margins or Mohs micrographic surgery is recommended for treatment of the primary tumor regardless of tumor size. However, recent studies have suggested that Mohs may be a superior treatment method, as it ensures 100% superficial and deep margin assessment [15]. However, prospective randomized control trials are needed to fully evaluate this benefit. Mohs additionally has the cosmetic advantage of tissue sparing compared to wide local excision and does not require re-resections if a delayed pathologic read shows a positive margin as can occur with wide resection [16,17]. Lymph node dissection is also used in node-positive disease. Sentinel lymph node biopsy (SLNB) is recommended for clinically node-negative patients, and according to National Comprehensive Cancer Network (NCCN) guidelines can be done prior to, at the same time as, or after definitive excision of the primary lesion. MCC is generally chemotherapy-sensitive, but survival benefit has not been shown, and recurrence often develops shortly after therapy. Therefore, it is generally used palliatively [18]. More recent research has shown the effectiveness of immunotherapy at treating MCC, such as with pembrolizumab and avelumab, PD(L)-1 inhibitors, both of which are FDA-approved for treatment of advanced disease [19,20].

Due to the multiple treatment modalities, patients often require coordination with a wide variety of specialists, including those from the fields of dermatology, medical oncology, surgical or head and neck oncology, and radiation oncology. This can be burdensome to patients, as initiation of treatment can require numerous medical appointments. Additionally, studies have shown that lower income status, uninsured or underinsured status, and further travel distances to medical appointments are associated with reduced MCC survival rates [21,22,23]. The large number of visits might contribute to this disparity. Numerous visits can increase the financial burden due to transportation and lodging costs, as well as missed work. In fact, studies have shown that the average time between SLNB and surgery is around 6 days, which could represent 6 days of missed work and lodging costs [24]. Therefore, we propose that limiting the number of visits may help improve patient outcomes and satisfaction. Our study seeks to address some of these barriers to multidisciplinary care.

The purpose of this study is to compare the outcomes of the traditional diagnosis and initial treatment of MCC to that of Cleveland Clinic Foundation’s (CCF) multidisciplinary “Merkel cell carcinoma in a day” research protocol (MIAD). Using the coordination of the Mohs surgical team, nuclear medicine, sentinel node biopsy (SLNB) and reconstructive surgery teams, all procedures are performed on the same day, reducing the number of visits and travel time for patients.

## 2. Materials and Methods

### 2.1. Study Design

**Figure 1:**
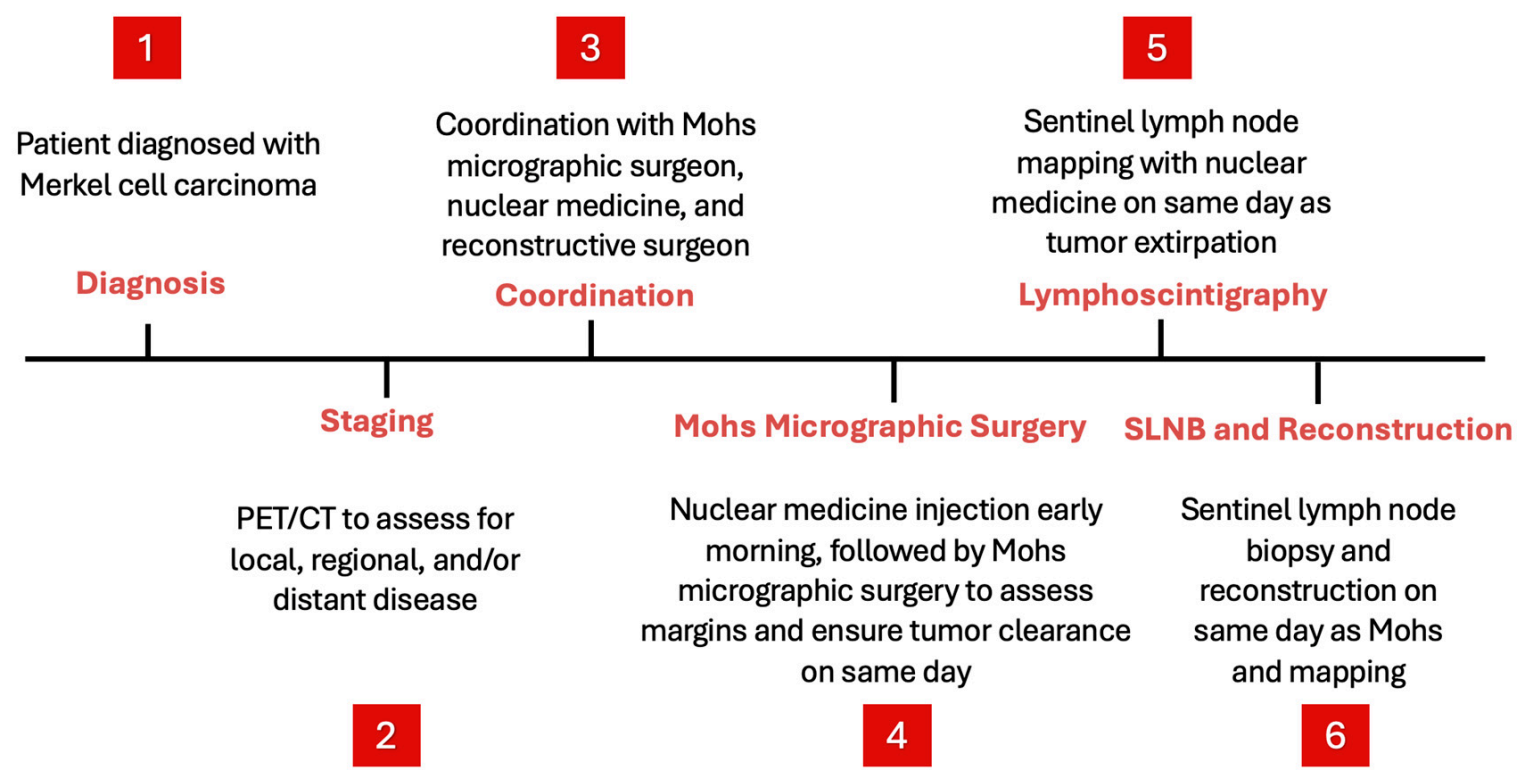
Timeline for MIAD protocol. After diagnosis is made and staging demonstrates need for SLNB, coordination is performed among the departments to reduce the number of hospital visits. Steps four, five, and six occur on the same day, where a patient receives their injection with nuclear medicine in the morning, followed my Mohs micrographic surgery, then finally SLNB and reconstruction.

This study is a retrospective, single-center cohort study. Patients who received treatment for MCC at CCF from 1 January 2012 to 21 June 2024 were included in the study. Additionally, specimen information was collected through surgery logs and pathology queries. Patients were excluded from the study if records of the surgical timing or procedure type were incomplete. Patients were sorted into the MIAD group if the SLNB and primary surgical excision of the lesion were completed within 24 h of each other (Figure 1) based on retrospective chart review. Those with more than 24 h between procedures were categorized as the “traditional protocol” group. Eligibility criteria for the MIAD group included: biopsy-proved MCC, a candidate for SLNB, tumor amenable to same-day Mohs and reconstruction, patient able to tolerate a long single day, nuclear medicine and plastics slot availability for the procedure. Assignment was not randomized and reflected clinical judgment plus scheduling feasibility. Additionally, patients were stratified into node-negative at presentation and node-positive at presentation. All clinically node-negative and PET CT-negative patients underwent SLNB. IRB approval was obtained for this study. Personal health data was collected for patients diagnosed with or treated for MCC at CCF or any of its associated satellite hospitals. Data was securely stored in the REDCap database. Access to registry and data collection was limited to those study members included on the IRB approval.

### 2.2. Data Collection

**Figure 2:**
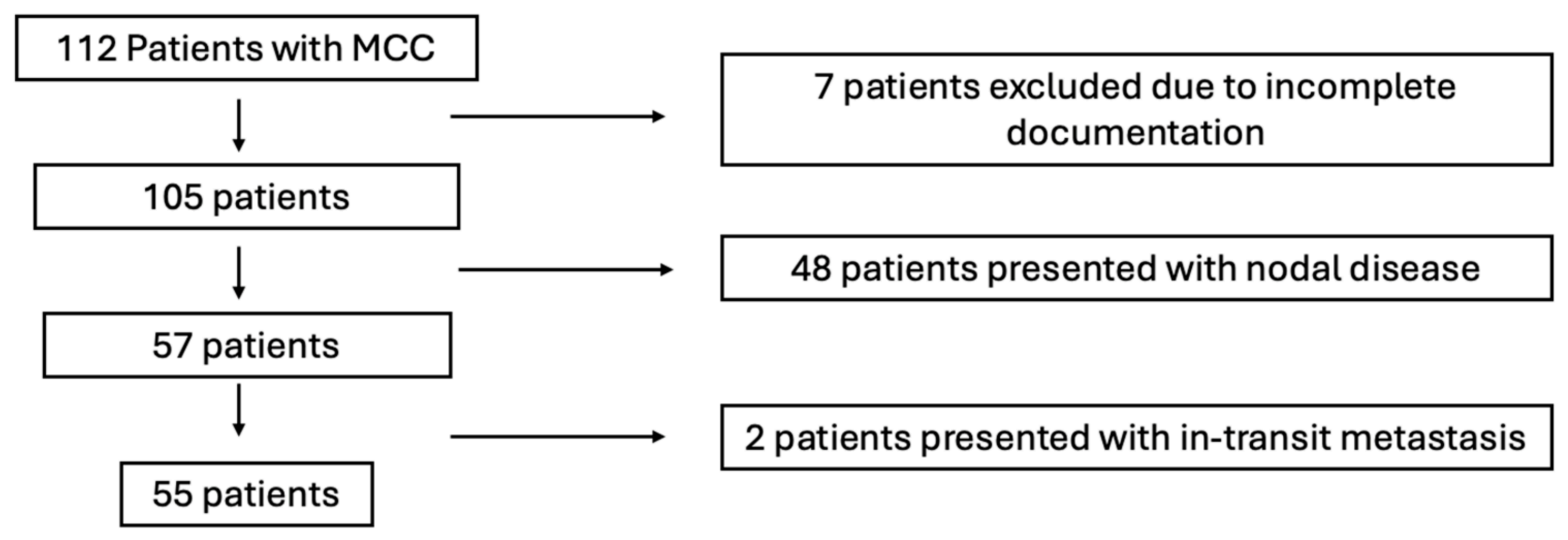
Calculation of total patients for outcome measures. Patients with a given outcome, or a more advanced one, at the time of presentation were excluded from the at-risk denominator for that outcome. In total, 105 patients were included in the study. Forty-eight patients presented with nodal disease; therefore, only 57 patients were included in the measurement of the occurrence of that outcome. Two patients presented with in-transit metastasis; therefore, only 55 patients were included in the measurement of the occurrence of that outcome or the outcome of local recurrence.

Clinical data was collected through the electronic medical record (EMR) including age, gender, race, and immunosuppression status as well as primary tumor characteristics including location (head and neck vs. other), clinical diameter, depth of invasion, and lymphovascular invasion. Treatment information collected included type of surgery performed, SLNB results, and the use of systemic therapies such as chemotherapy, immunotherapy, or radiation. Outcome measures included recurrence, disease-specific death, and complication rates. For outcome measures analysis was performed only on patients who did not have these outcomes or a more severe outcome at the time of presentation. Therefore, those with local recurrence, in-transit metastasis, nodal metastasis, or distant metastasis represent only those who developed those conditions following initial surgical management of the cancer. Recurrence is the first event of that type following completion of initial surgical management; progression of disease present at diagnosis was not counted as recurrence and those patients were excluded from the relevant denominator (Figure 2).

### 2.3. Analysis

Categorical variables are summarized as frequencies with percentages and were compared across groups using Pearson’s chi-squared test or Fisher’s exact test. Continuous measures were evaluated for normality using histograms and Shapiro–Wilk tests. Normally distributed measures were described using means with standard deviations and compared using two-sample *t*-tests, while other continuous measures were described using medians with quartiles and were compared using Wilcoxon rank sum tests. Competing risk regression survival analysis was used to compare features on outcomes. Models were univariable only. Fine–Gray sub-distribution hazard ratios with 95% confidence intervals are reported. Analysis was conducted on complete cases without imputation and denominators are reported for each variable. Analysis was performed using R software (version 4.3; Vienna, Austria). A significance level of 0.05 was used for all tests.

## 3. Results

A total of 112 records of patients with MCC were identified during the study timeframe. Seven records lacked documentation of the surgery type, resulting in 105 patients included in the final analysis.

Overall, the mean age of the cohort was 73 years; 72% were male and 96% identified as white; and 16% were immunosuppressed prior to their diagnoses (Table 1). Of the patients included, 67 patients received the traditional protocol, while 38 patients received the MIAD protocol. The median time between diagnoses of the cancer and treatment was 33 days; however, 46 of the 105 patients underwent treatment less than 30 days after diagnosis (Table 1). Diameter data was missing in 31/105 patients (30%). Lymphovascular invasion was not mentioned in 27 of the records (26%). Extracapsular extension data was missing for 73 patients (69.5%). Due to incomplete data on the primary tumor (clinical diameter and depth), AJCC staging was unable to be determined for the majority of tumors in both subgroups.

When stratified by surgery protocol, the two groups were demographically similar. We did not detect a difference in the age, gender, race, and immunosuppression status of the MIAD and traditional protocol groups (*p* > 0.25). Immune status was not available for one patient. The locations of the primary tumors were comparable, with 26 of 67 (39%) tumors presenting on the head and neck in the traditional group, compared to 19 of 38 (50%) in the MIAD group. However, those who received the MIAD protocol had smaller tumors (1.6 cm vs. 2.6 cm *p* = 0.060) and were less likely to have lymphovascular invasion than those in the traditional group (19% vs. 55% *p* = 0.002) (Table 2). Radiation use was numerically higher in the MIAD group (71% vs. 52%, *p* = 0.060). The use of systemic therapy was numerically similar (*p* = 0.74). Instances of complications from the primary surgical treatment of the MCC were very few among both groups. Only six patients in the traditional group experienced complication(s), and only one patient in the MIAD group. The types of complications include re-hospitalization, scar revision, infection, other, or any event that required additional intervention (Table 2).

Both groups had numerically similar median years of treatment (*p* = 0.33), and the treatment was proportioned through the study duration (*p* = 0.12). There was no detected difference in the time to treatment between the two groups, with a median of 30 days for the traditional group and a median of 34 days for the MIAD group (*p* = 0.14). A total of 51% of patients in the traditional group received treatment within 30 days and 32% of patients in the MIAD group (*p* = 0.057). The median follow-up time between the MIAD group and the traditional group was 18 and 23 months respectively (ranges 0–179 and 2–124) (Table 2). MCC recurs mostly within 3 years, and our median follow-up is 18–23 months; therefore, a meaningful fraction of patients had insufficient follow-up to capture recurrence [13]. The rate of local recurrence for patients with available recurrence data was 7.3% (4/55) (Table 1). Rates of local recurrence, in-transit metastasis, distant metastasis, and disease-specific death were numerically similar among the two groups (*p* = 0.71, 0.3, 0.33, 0.63) (Table 3). Given four to six events per outcome, only very large effect sizes were detectable. The MIAD group had a statistically significantly higher incidence of nodal metastasis compared to the traditional protocol of 4/23 and 2/34, respectively (*p* = 0.039). The wide confidence interval indicates the magnitude of this association is imprecisely estimated. No adjustment was made for multiple comparisons across the five outcomes analyzed. Additionally, the MIAD patients were node-negative at presentation by design; therefore, they are differentially at risk for subsequent nodal events.

## 4. Discussion

Consistent with the previous literature, most patients in the study fit the classic description of an individual with MCC: elderly white men [9]. Therefore, strategies used to diagnose and treat this subset of patients may be applicable to most MCC cases.

Rates of local recurrence, in-transit metastasis, distant metastasis, and disease-specific death were numerically similar between groups, with no statistically significant differences detected. A higher incidence of nodal metastasis was observed in the MIAD group (HR 4.94, 95% CI 1.08–22.6, *p* = 0.039), based on six events. These comparisons should be interpreted with caution. With four to six events per outcome the study had limited power to detect clinically meaningful differences; median follow-up was 18–23 months in a disease in which most recurrences occur within three years, and the groups differed at baseline in tumor diameter and lymphovascular invasion, which were not adjusted for. These data describe the observed experience of this cohort and cannot establish whether oncologic outcomes under the MIAD protocol are comparable to those under traditional management.

There was no statistical difference in the spread of the cases across the study period of 2012–2024 (*p* = 0.12). The systemic therapies avelumab and pembrolizumab were approved for the treatment of MCC in 2017 and 2018 respectively [19,20]. The introduction of these drugs would have certainly changed the outcomes of patients within the study. However, since the patients are proportioned throughout the study, this confounding effect should be minimized. Additionally, both the MIAD and the traditional protocols were able to be performed in similar timeframes between diagnosis of MCC and initial treatment (34 vs. 30 days, *p* = 0.14). The MIAD group did have a statistically significantly smaller portion treated within the target 30 days compared to the traditional group (12% vs. 34%). Consolidating three departments into one day required aligning multiple schedules, which can add front-end delay. However, we hypothesize that this can also reduce the number of visits for the patients. Our protocol was designed to reduce the number of visits to the clinic, which can often involve traveling long distances. Further study is needed to assess how and if the number of visits were reduced with this protocol. Overall, the results of this study showed promise.

The previous literature has shown that multidisciplinary care and coordination of cancer treatment have improved patient satisfaction for other diagnoses [25,26]. Additionally, multidisciplinary, coordinated care has been shown to decrease the number of visits for patients with advanced SCC at similarly large academic medical centers [27]. This is most realistic at large academic medical centers, where departments can easily communicate and patients can feasibly travel between departments in a single day. Our study did not observe evidence of worse outcomes, although the study was not powered to exclude a difference. Therefore, we urge other centers to attempt this type of care for their patients, especially at institutions with resources like those of CCF.

While some patients in the traditional protocol group underwent Mohs micrographic surgery, a large portion underwent wide local excision of their tumor. NCCN guidelines do not advocate for one surgical method over the other; however, recent studies have suggested Mohs may be a superior method, as it allows for evaluation of 100% of the deep and peripheral tissue margins [15,16,28]. Our study did not compare clinical outcomes among those that underwent WLE vs. Mohs. However, a coordination of care protocol, like the one in our study, may lead to increased availability of Mohs micrographic surgery. The previous literature has shown that the majority of Mohs surgeons are in urban areas [29]. As a result, those in rural areas might have more difficulty accessing this type of care. If care was coordinated, patients would only travel to large urban medical centers once. This may allow for a larger portion of those individuals to undergo Mohs surgery, which may improve outcomes. However, further studies are needed to assess the efficacy of Mohs surgery compared to WLE. Additionally, studies have demonstrated that patients treated at large-volume academic medical centers exhibit improved outcomes compared to patients treated at lower-volume centers [30]. By limiting the number of visits needed to receive treatment at large urban centers, patients may be more easily able to access superior care.

The major limitation of this study is the sample size and lack of power to demonstrate equivalence. The small sample size also prevents the use of multi-variable analysis, which would have allowed for further characterizations of the differences. Another limitation is that the MIAD group had systematically more favorable primary tumors. This follows directly from the eligibility criteria (smaller tumors amenable to same-day Mohs plus reconstruction); therefore, confounding by indication cannot be excluded, and outcome comparisons are therefore descriptive rather than causal. Additionally, this protocol was implemented at a single large academic medical center, and it remains uncertain whether similar feasibility and outcomes would be achieved at other smaller, less resourced institutions. However, our patient population is representative of the classic MCC patient; therefore, similar results may be seen at other centers. Further study is needed to evaluate the implementation of the MIAD protocol at other centers. Finally, this study was performed retrospectively, which limits the design. However, prospective studies are difficult to perform on rare diagnoses such as MCC; therefore, these results are still relevant to clinical practice.

## 5. Conclusions

The “Merkel cell carcinoma in a day” protocol is logistically feasible. This study was not designed or powered as an equivalence of noninferiority trial and the confidence intervals do not exclude clinically meaningful differences in either direction. However, our study suggests that this is feasible in clinical practice. Coordinating care for patients has been shown to be highly beneficial in numerous studies of other diagnoses [25,26,27]. Therefore, MIAD might improve patient satisfaction and reduce barriers to care, particularly at similarly large multidisciplinary medical centers. Further prospective studies on patient outcomes and satisfaction need to be conducted to assess for these findings.

## Figures and Tables

**Table 1:** Overall summaries.

Characteristic	Total *n*	Summary
Age (years), mean (SD)	105	73 (11)
Gender, *n* (%)	105	
Female		29 (28)
Male		76 (72)
Race, n (%)	105	
Black		2 (1.9)
Hispanic		2 (1.9)
White		101 (96)
Immunosuppressed prior to Merkel cell Dx, *n* (%)	104	17 (16)
Presentation: primary tumor, *n* (%)	105	104 (99)
Presentation: local recurrence, *n* (%)	105	0 (0)
Presentation: in-transit metastasis, *n* (%)	105	2 (1.9)
Presentation: nodal disease, *n* (%)	105	48 (46)
Presentation: distant metastasis, *n* (%)	105	1 (1.0)
Location of primary, *n* (%)	105	
Other		60 (57)
Head/neck		45 (43)
Primary clinical tumor diameter, median (IQR)	74	2.00 (1.10, 3.50)
Primary tumor depth of invasion, *n* (%)	105	
Dermis		12 (11)
Fascia		1 (1.0)
Muscle		3 (2.9)
Not reported/not specified		74 (70)
Subcutaneous fat		15 (14)
Lymphovascular invasion, *n* (%)	78	33 (42)
Nodal involvement, *n* (%)	105	
No/unknown		57 (54)
Yes		48 (46)
Extracapsular extension, *n* (%)	32	12 (38)
Surgery type, *n* (%)	105	
Excision		62 (59)
Mohs		43 (41)
Lymphadenectomy, *n* (%)	105	85 (81)
Protocol: Merkel cell carcinoma in a day, *n* (%)	105	38 (36)
Time to treatment (days), median (IQR)	105	33 (20, 44)
Treatment within 30 days, *n* (%)	105	46 (44)
Surgery treatment, *n* (%)	105	
Surgery alone		42 (40)
Surgery + radiation/systemic		63 (60)
Local recurrence, *n* (%)	55 ^†^	4 (7.3)
In-transit metastasis, *n* (%)	55 ^†^	5 (9.1)
Nodal metastasis, *n* (%)	57 ^†^	6 (11)
Distant metastasis, *n* (%)	104 ^†^	17 (16)

^†^ See Figure 2 for calculation of Total N based on protocol described in Section 2.2.

**Table 2:** Patient characteristics by protocol.

Characteristic	*n*	Traditional *n*= 67	Merkel Cell Carcinoma in a Day *n* = 38	*p*-Value ^1^
Age (years), mean (SD)	105	74 (11)	72 (9)	0.25
Gender, ***n*** (%)	105			0.49
Female		17 (25)	12 (32)	
Male		50 (75)	26 (68)	
Race, ***n*** (%)	105			0.40
Black		2 (3.0)	0 (0)	
Hispanic		2 (3.0)	0 (0)	
White		63 (94)	38 (100)	
Immunosuppressed prior to Merkel cell Dx, ***n*** (%)	104	9 (14)	8 (21)	0.32
Location of primary, ***n*** (%)	105			0.27
Other		41 (61)	19 (50)	
Head/neck		26 (39)	19 (50)	
Primary clinical tumor diameter, median (IQR)	74	2.6 (1.1, 4.5)	1.6 (1.0, 2.0)	0.060
Treatment year, median (IQR)	105	2018 (2015, 2021)	2020 (2017, 2021)	0.33
Treatment year, ***n*** (%)	105			0.12
2012–2014		16 (24)	7 (18)	
2015–2018		22 (33)	8 (21)	
2019–2021		15 (22)	17 (45)	
2022–2024		14 (21)	6 (16)	
Follow-up time, median (IQR) (months)		23 (6, 64)	18 (9, 43)	
Follow-up time, range (months)		0–179	2–124	
Time to treatment (days), median (IQR)	105	30 (14,45)	34 (27,41)	0.14
Treatment within 30 days, ***n*** (%)	105	34 (51)	12 (32)	0.057
SLNB	105	61 (91)	38 (100)	0.085
Primary tumor depth of invasion, ***n*** (%)	105			0.63
Dermis		6 (9.0)	6 (16)	
Fascia		1 (1.5)	0 (0)	
Muscle		3 (4.5)	0 (0)	
Not reported/not specified		47 (70)	27 (71)	
Subcutaneous fat		10 (15)	5 (13)	
Lymphovascular invasion, ***n*** (%)	78	28 (55)	5 (19)	0.002
Treatment after diagnosis:				
Radiation, ***n*** (%)	105	35 (52)	27 (71)	0.060
Systemic, ***n*** (%)	105	7 (10)	3 (7.9)	0.28
Surgical complications	105			0.74
Yes		6 (8.9)	1 (2.6)	
No		56 (84)	37 (97)	
Not reported		5 (7.5)	0 (0)	
Surgical complications type	105			
Re-hospitalization, ***n*** (%)		1 (1.5)	0 (0)	>099
Scar revision, ***n*** (%)		1 (1.5)	0 (0)	>0.99
Infection, ***n*** (%)		2 (3.0)	0 (0)	0.53
Any intervention, ***n*** (%)		1 (1.5)	0 (0)	>0.99
Other, ***n*** (%)		4 (6.0)	1 (2.6)	0.65

^1^ Welch’s two-sample *t*-test; Pearson’s chi-squared test; Fisher’s exact test; Wilcoxon rank sum test.

**Table 3:** Unadjusted outcomes by surgery protocol.

Event	Protocol	Total *n*	Event *n*	HR (95% CI)	*p*-Value
Local Recurrence					
	Traditional	32	2	Reference	
	MIAD	23	2	1.44 (0.21, 9.88)	0.71
In-Transit Metastasis					
	Traditional	32	4	Reference	
	MIAD	23	1	0.33 (0.04, 2.70)	0.30
Nodal Metastasis					
	Traditional	34	2	Reference	
	MIAD	23	4	4.94 (1.08, 22.6)	0.039
Distant Metastasis					
	Traditional	66	13	Reference	
	MIAD	38	4	0.57 (0.19, 1.75)	0.33
Disease-Specific Death					
	Traditional	67	12	Reference	
	MIAD	38	7	1.26 (0.49, 3.23)	0.63

Abbreviations: CI = confidence interval, HR = hazard ratio.

## Data Availability

The data presented in this study are available on request from the corresponding author. The data is not publicly available due to patient privacy and inclusion of protected personal health information in the data set.

## Abbreviations

The following abbreviations are used in this manuscript:

MCC	Merkel cell carcinoma
OS	Overall survival
MCV	Merkel cell polyomavirus
Rb	Retinoblastoma protein
LTA	Large T antigen
STA	Small T antigen
SCC	Squamous cell carcinoma
TTF-1	Thyroid Transcription Factor 1
AJCC	American Joint Committee on Cancer
SLNB	Sentinel lymph node biopsy
NCCN	National Comprehensive Cancer Network
CCF	Cleveland Clinic Foundation
MIAD	Merkel cell carcinoma in a day
EMR	Electronic Medical Record
